# Inflammasome Activation Induced by Perfringolysin O of *Clostridium perfringens* and Its Involvement in the Progression of Gas Gangrene

**DOI:** 10.3389/fmicb.2019.02406

**Published:** 2019-10-25

**Authors:** Kiyonobu Yamamura, Hiroshi Ashida, Tokuju Okano, Ryo Kinoshita-Daitoku, Shiho Suzuki, Kaori Ohtani, Miwako Hamagaki, Tohru Ikeda, Toshihiko Suzuki

**Affiliations:** ^1^Department of Bacterial Pathogenesis, Infection and Host Response, Graduate School of Medical and Dental Sciences, Tokyo Medical and Dental University, Tokyo, Japan; ^2^Department of Bacteriology and Bacterial Infection, Division of Host Defense Mechanism, Tokai University School of Medicine, Isehara, Japan; ^3^Department of Oral Pathology, Graduate School of Medical and Dental Sciences, Tokyo Medical and Dental University, Tokyo, Japan

**Keywords:** *Clostridium perfringens*, gas gangrene, inflammasome, caspase-1, perfringolysin O

## Abstract

*Clostridium perfringens* (*C. perfringens*) is Gram-positive anaerobic, spore-forming rod-shaped bacterial pathogen that is widely distributed in nature. This bacterium is known as the causative agent of a foodborne illness and of gas gangrene. While the major virulence factors are the α-toxin and perfringolysin O (PFO) produced by type A strains of *C. perfringens*, the precise mechanisms of how these toxins induce the development of gas gangrene are still not well understood. In this study, we analyzed the host responses to these toxins, including inflammasome activation, using mouse bone marrow-derived macrophages (BMDMs). Our results demonstrated, for the first time, that *C. perfringens* triggers the activation of caspase-1 and release of IL-1β through PFO-mediated inflammasome activation via a receptor of the Nod-like receptor (NLR) family, pyrin-domain containing 3 protein (NLRP3). The PFO-mediated inflammasome activation was not induced in the cultured myocytes. We further analyzed the functional roles of the toxins in inducing myonecrosis in a mouse model of gas gangrene. Although the myonecrosis was found to be largely dependent on the α-toxin, PFO also induced myonecrosis to a lesser extent, again through the mediation of NLRP3. These results suggest that *C. perfringens* triggers inflammatory responses via PFO-mediated inflammasome activation via NLRP3, and that this axis contributes in part to the progression of gas gangrene. Our findings provide a novel insight into the molecular mechanisms underlying the pathogenesis of gas gangrene caused by *C. perfringens*.

## Introduction

*Clostridium perfringens* is commonly isolated from the environment (e.g., soil), and also from human and animal intestines as a component of the normal flora (Songer, 1996). *C. perfringens* has been classified into five groups (types A to E) according to their production of four major toxins, namely, α (CPA), β (CPB), ε (ETX), and ι (ITX) toxin (Uzal et al., 2010). Moreover, the bacteria can also produce up to 16 other toxins in various combinations, including perfringolysin O (PFO, also called θ-toxin), enterotoxin (CPE), and beta2 toxin (CPB2) (Uzal et al., 2010). Type A *C. perfringens* is the causative strain for the majority of human infections, including gas gangrene. Gas gangrene is characterized by severe muscle tissue destruction (myonecrosis), gas production, and massive local edema (Bryant and Stevens, 2010). The α-toxin and PFO produced by the type A strains are the major virulence factors of *C. perfringens*. *In vivo* studies using murine myonecrosis models and mutant strains lacking α-toxin and PFO have provided strong evidence for the roles of these toxins in the progression of myonecrosis (Awad et al., 1995, 2001; Ellemor et al., 1999). However, the precise mechanisms underlying the toxin-mediated myonecrosis in gas gangrene are still unclear.

In regard to the mechanism of induction of myonecrosis by *C. perfringens*, α-toxin and PFO produced by the bacteria synergistically upregulate the expressions of adhesion molecules on the surfaces of vascular endothelial cells and neutrophils (Bryant and Stevens, 1996; Bunting et al., 1997), which leads to platelet-leukocyte aggregation and reduction of microvascular perfusion. The local vascular occlusion caused thus has been implicated in the tissue ischemia and consequent myonecrosis caused by the infection (Bryant et al., 2000a, b, 2006; Ochi et al., 2002; Hickey et al., 2008). Furthermore, it has also been suggested that the bacteria subvert the host immune responses by inhibiting leukocyte entry into the infected tissues, namely, by causing leukostasis (Ellemor et al., 1999; Bryant et al., 2000b). On the other hand, upregulation by the bacterium of several inflammation-associated genes has also been reported. PFO activates the expressions of the cytokines TNF-α and IL-6 in macrophages (Park et al., 2004), and α-toxin, but not PFO, has been demonstrated to induce IL-8 production in human vascular endothelial cells (Bryant and Stevens, 1996) and in a lung adenocarcinoma epithelial cell line A549 (Oda et al., 2012). Injection of α-toxin into mice elevated the serum levels of TNF-α, IL-1β, and/or IL-6 (Oda et al., 2008). How best to interpret the apparent reciprocal functions of α-toxin and PFO in activating/subverting the inflammatory responses of the host to *C. perfringens* infection remains controversial and still under debate.

A recent study in which transcriptional analysis of the infected muscle tissue of mice was performed by RNA sequencing showed that a number of inflammation-associated genes were upregulated in regions of myonecrosis induced by *C. perfringens* (Low et al., 2018), including genes of the chemokine family CXCL2, and of proinflammatory cytokines such as IL-1β and IL-6. Components of inflammasome activation, including NLRP3, were also up-regulated. The inflammasomes are known to regulate the production of some inflammatory cytokines. Activation of inflammasomes results in conversion of caspase-1 to its active form, which, in turn, proteolytically processes pro-IL-1β and pro-IL-18 to produce active cytokines. The family of NLRs finely regulates caspase-1 activation in response to extracellular stimuli (Higa et al., 2013; Lamkanfi and Dixit, 2017). The upregulation of genes associated with inflammasome activation, such as NLRP3, suggested the possibility, although accumulated data had not yet demonstrated the actual inflammasome activation and cytokine production in tissues infected by clostridial strains (Low et al., 2018).

In this paper, we investigated the induction of inflammasome activation by *C. perfringens* in infected mouse macrophages. The bacteria trigger caspase-1 activation and consequently, IL-1β release. PFO, but not α-toxin, was found to be an essential factor for triggering inflammasome activation via the mediation of NLRP3. The PFO-mediated inflammasome activation was not induced in cultured mouse skeletal myocytes. Furthermore, we first demonstrated that the myonecrosis induced by PFO was dependent on NLRP3, suggesting that the PFO produced by *C. perfringens* induces myonecrosis in infected muscle tissues via NLRP3-mediated inflammasome activation.

## Materials and Methods

### Ethics Statement

All animal studies were performed in strict compliance with the Guidelines for Animal Experimentation of the Japanese Association for Laboratory Animal Science. All protocols were approved by the Institutional Animal Care and Use Committee of Tokyo Medical and Dental University (approval number: A2019-019A). The experimental protocols covering the use of a Living Modified Organism, including bacterial mutants and gene-knockout mice, were approved by the Genetically Modified Organisms Safety Committee of Tokyo Medical and Dental University (approval number: G2018-021C2). The handling of *C. perfringens* and *Salmonella enterica* strains under biosafety level 2 condition was approved by the Safety Control Committee for Pathogenic Microbes of Tokyo Medical and Dental University (approval number: M22019-004).

### Bacterial Strains

The wild-type (WT) *C. perfringens* strain 13 was used in this study (Shimizu et al., 2002). Isogenic *C. perfringens* mutants, namely, Δ*plc*, lacking the α-toxin, Δ*pfo*, lacking PFO, and a double-mutant of these genes (Δ*plc*Δ*pfo*), were constructed using allele replacement strategies and suicide vectors (Shimizu et al., 1994; Takehara et al., 2016). One strain of another type of *C. perfringens*, ATCC13124, was also used in this study (Mollby et al., 1976). The bacterial strains were grown in Gifu anaerobic medium (GAM) broth (Nissui Seiyaku Co., Ltd., Tokyo). *S. enterica* serovar Typhimurium Δ*sseD*, an SPI-2-deficient mutant (Liu et al., 2014), was grown in Luria-Bertani broth.

### Mice, Preparation of Macrophages, and Cell Line

C57BL/6 mice were purchased from Japan SLC (Tokyo, Japan) as WT mice. NLRP3-deficient (*Nlrp3*^–/^*^–^*) and ASC (*Asc*^–/–^ or *Pycard*^–/–^)-deficient mice with a C57BL/6 background were housed in a specific-pathogen-free facility. BMDMs were prepared from the femurs of the above-mentioned mice and cultured for 5–6 days in 10% FBS-RPMI 1640 (Sigma) supplemented with 30% mouse L-cell supernatant. L-cell supernatant containing macrophage colony stimulating factor (M-CSF) was prepared by cultivating mouse L929 cells for 10 days.

### Reagents

Ultrapure LPS and nigericin were purchased from Invivogen and Sigma-Aldrich, respectively. The following antibodies were purchased commercially: rabbit anti-NLRP3 (cryo-2; Adipogen, AG-20B-0014), rabbit anti-ASC (AL177; Adipogen, AG-25B-0006), mouse anti-caspase-1 (p20) (Casper-1; Adipogen, AG-20B0042), mouse anti-caspase-1 (p10) (Casper-2; Adipogen, AG-20B0044), goat anti-mouse IL-1β antibody (R&D, AF-401-NA), anti-gasdermin D (Cell Signaling, #93709), anti-actin (clone 4; Merck Millipore, #MAB1501), anti-F4/80 (Cell Signaling, #70076).

### Bacterial Infection, LDH Assay, and ELISA

Bone marrow-derived macrophages were seeded at a density of 0.35 × 10^6^ cells per well in 24-well plates containing 10% FBS-RPMI 1640. The LPS (100 ng/ml, 2 h)-primed cells were infected with *C. perfringens* at a multiplicity of infection (MOI) of 2.5 (ATCC13124) or 25 (strain 13) per cell. The plates were incubated at 37°C. At the indicated times after infection, lactate dehydrogenase (LDH) activity in the culture supernatants was measured using a CytoTox 96 assay kit (Promega, Madison, WI, United States), in accordance with the manufacturer’s protocol. The following formula was used to calculate the amount of LDH released: [(OD490 sample release-OD490 negative control release)/(OD490 positive control release-OD490 negative control release)] ×100, where OD490 negative control release represents the amount of LDH released into the culture supernatant from uninfected cells and OD490 positive control release represents the amount of LDH released after lysis of the uninfected cells. Cytokines released in the culture supernatants were quantified by ELISA (eBioscience). The endpoint of the experiments was defined as 1 h post-infection (hpi), because *C. perfringens* are extracellular pathogens and multiply quickly in cell culture media.

### Immunoblotting

Bone marrow-derived macrophages were seeded at a density of 1.3 × 10^6^ cells per well in 6-well plates and infected with bacteria. The cells were lysed and mixed with the supernatant precipitated with 10% trichloroacetic acid. The samples were subjected to 15% SDS-PAGE and immunoblotting.

### Differentiation of Mouse Myoblast C2C12 Cells and Infection

Mouse myoblasts C2C12 cells (RCB-0987, RIKRN BRC, Japan) were grown in Dulbecco’s Modified Eagle Medium (DMEM)-low glucose (Sigma) containing 10% FBS. For differentiation of myocytes (myotubes), the cells were cultured in DMEM-high glucose containing 2% horse serum for 5 days. The myoblasts or the differentiated myocytes were treated with LPS (1 μg/ml) or IFN-γ (100 ng/ml) for 24 to 72 h before infection with *C. perfringens* at MOI of 100.

### Mouse Infection for Creation of a Gas Gangrene Model

*Clostridium perfringens* strains were cultured anaerobically. A washed suspension (100 μl) of the bacterial cells in PBS containing 1 × 10^9^ CFU was injected intramuscularly into the thighs of 6-week-old male C57BL/6 mice, as described previously (Awad et al., 2001). Mice were sacrificed at 6 hpi and the muscle tissues of the thigh were harvested. The bacterial load was determined by plating dilutions of the tissue homogenates, and the cytokine levels in the muscle tissues were measured by ELISA. For histopathological examination, the muscle tissues were fixed in 10% formalin for HE staining and Gram staining. Immunostainings to detect macrophage lineage were performed using anti-F4/80 antibody, HRP-coupled anti-rabbit polymer Ig (Dako) and 3,3′-diaminobenzidine (DAB). For measuring the area of myonecrosis, we used a public-domain imaging software, ImageJ (version 1.52a^1^; National Institutes of Health, United States). First, entire muscle tissue part and entire myonecrosis part of the histological images of HE staining were circled and scaled. Then, the lesion area was divided by the total area to calculate the percent area affected by gas gangrene. In the selection of the lesion site, areas showing fragmentation and flocculation of the sarcolemma and no stainable nuclei, which are characteristic findings of gas gangrene, were selected, according to previous reports (Keese et al., 2003; Uzal et al., 2014). Areas of muscle tissue that showed only edema were not included in the lesion.

### Statistical Analysis

All data are presented as the means ± standard deviation of at least three determinations per experimental condition. All experiments were performed at least three times, and the representative results are shown in the figures. Statistical analyses were performed by one-way analysis of variance (ANOVA) followed by Tukey’s test or unpaired two-tailed Student’s *t*-tests. Differences at *p*-values of <0.05 were considered to be statistically significant.

## Results

### Infection With *C. perfringens* Induces Inflammasome Activation in Macrophages, and Consequently, Caspase-1 Activation, IL-1β Release and Pyroptosis

**FIGURE 1:**
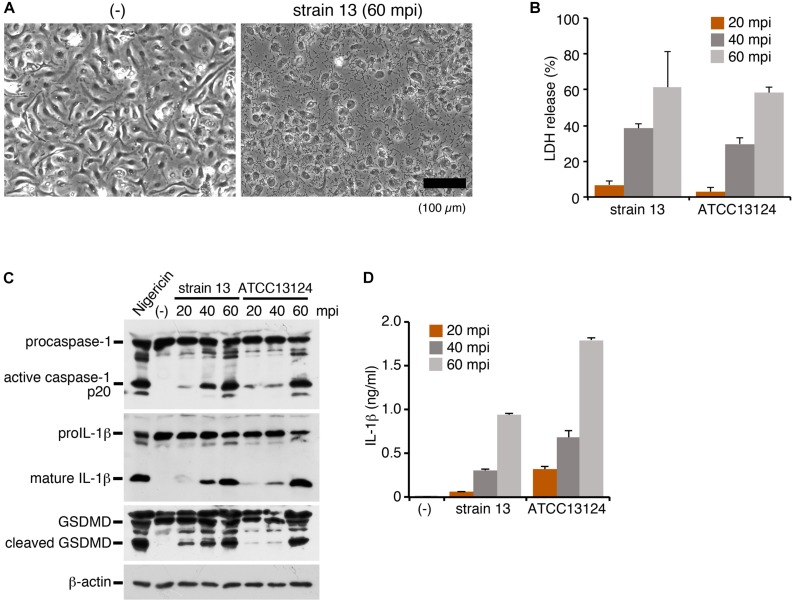
Infection with *Clostridium perfringens* induces inflammasome activation with caspase-1 activation, IL-1β release and pyroptosis in macrophages. BMDMs from C57BL/6 mice were primed with LPS (100 ng/ml) for 2 h and infected with *C. perfringens* (strain 13 or ATCC13124) for the indicated times, or treated with 10 μM nigericin for 30 min as a positive control. **(A)** Phase- contrast images of uninfected (-) or strain 13-infected BMDMs at 60 mpi. Bar, 100 μm. **(B)** Culture supernatants of the infected BMDMs were analyzed for LDH release at 20–60 mpi. **(C)** Activation of caspase-1, and cleavage of IL-1β or GSDMD in the infected BMDMs was analyzed by immunoblotting with anti-caspase-1 (p20), anti-IL-1β and anti-GSDMD antibody, respectively. The levels of β-actin as the loading control were also examined. **(D)** IL-1β release from the infected BMDMs into the culture supernatants at 20–60 mpi was analyzed by an ELISA. Data are presented as the means ± SD of triplicate samples.

To investigate the inflammasome activation in the host cells directed against *C. perfringens* infection, LPS-primed mouse BMDMs were infected with strain 13, a wild-type (WT) strain of *C. perfringens*. At 60 min post infection (mpi), the infected cells caused necrotic cell death with loss of membrane integrity (Figure 1A). Lactate dehydrogenase (LDH), one of cytoplasmic enzymes, was released from the infected BMDMs, confirming the necrotic cell death of the macrophages (Figure 1B). Inflammasome activation by infection with *C. perfringens* was examined by immunoblot analysis using anti-caspase-1 and anti-IL-1β, as well as by ELISA for IL-1β. Rapid caspase-1 activation and cleavage/release of mature IL-1β were observed in the macrophages infected by the clostridial strain 13 (Figures 1C,D). Gasdermin D (GSDMD), the key molecule that triggers pyroptosis (Shi et al., 2017), was also cleaved and converted to its active form (Figure 1C). We next examined the host inflammatory responses following infection of macrophages with another WT strain, ATCC13124. As shown in Figures 1A,D, ATCC 13124 induced pyroptosis to the same degree as strain 13, but at a lower MOI (2.5) as compared to strain 13 (25). Taken together, these results indicated that *C. perfringens* infection induces inflammasome activation and pyroptosis, a form of proinflammatory cell death of macrophages, with release of IL-1β.

To clarify how LPS priming affects inflammasome activation in cells with *C. perfringens* infection, we examined the activation level of caspase-1 and IL-1β in the infected BMDMs primed or not primed with LPS. Caspase-1 activation was readily detected, to comparable degrees, in both the presence and absence of LPS priming of the cells (Supplementary Figure S1A). On the other hand, cleavage/release of IL-1β was scarcely detectable in the absence of LPS priming, because of the low expression level of the precursor of IL-1β (pro IL-1β) (Supplementary Figures S1A,B). The data suggest that priming with LPS is not necessary for induction of caspase-1 activation by *C. perfringens*. On the other hand, the small amounts of other cytokine (TNF-α but not IL-6) were released from infected macrophages (Supplementary Figure S1C), suggesting that bacterial components such as peptidoglycan may be able to stimulate cellular signaling to compensate for pretreatment with LPS (Akira et al., 2006).

### The Inflammasome Activation by *C. perfringens* Is Mediated by the Pore-Forming Toxin, Perfringolysin O

**FIGURE 2:**
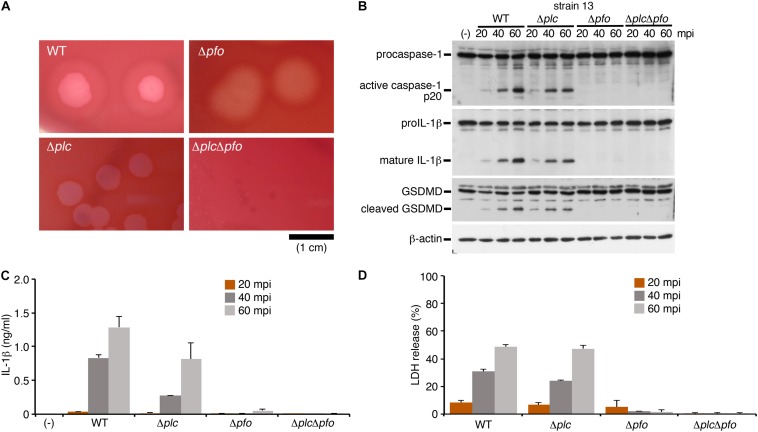
The inflammasome activation induced by *Clostridium perfringens* is mediated by the pore-forming toxin, perfringolysin O (PFO). BMDMs were primed with LPS for 2 h, and infected with WT *C. perfringens* (strain 13) and/or any of its isogenic mutants, Δ*plc* (α-toxin mutant), Δ*pfo* (PFO mutant) and Δ*plc*Δ*pfo* (double-mutant of α-toxin and PFO). **(A)** 1.5% (w/v) agar plates containing brain heart infusion (BHI) medium and 5% (v/v) defibrinated sheep blood show different degrees of hemolysis induced by the *C. perfringens* strains. Bar, 1 cm. **(B)** Activation of caspase-1, and cleavage of IL-1β or GSDMD in the infected BMDMs was analyzed by immunoblotting with anti-caspase-1 (p20), anti-IL-1β and anti-GSDMD antibody, respectively. **(C)** IL-1β release from the infected BMDMs into the culture supernatants at 20–60 mpi was analyzed by an ELISA. **(D)** Culture supernatants from infected BMDMs were analyzed for LDH release at 20–60 mpi. Data are presented as the means ± SD of triplicate samples.

To identify bacterial factors that trigger inflammasome activation, we focused on the clostridial toxins secreted from *C. perfringens* type A, namely, α-toxin and perfringolysin O (PFO). While α-toxin is phospholipase C, which modifies cell membranes by degrading phosphatidylcholine and sphingomyelin, the biological activity of PFO is to induce pore formation on the cell membrane of the host cells (Uzal et al., 2014). The bacterial colonies were surrounded by a wide zone of incomplete hemolysis with a narrow zone of complete hemolysis on sheep blood agar plates. The former (incomplete hemolysis) was mediated by α-toxin, while the latter (complete hemolysis) was induced by PFO, as seen in the mutants grown on blood agar. To examine which toxins produced by *C. perfringens* induce inflammasome activation, the isogenic mutants Δ*plc* (α-toxin mutant), Δ*pfo* (PFO mutant) and Δ*plc*Δ*pfo* (double-mutant of α-toxin and PFO) were constructed using allele replacement strategies and suicide vectors (Figure 2A; Shimizu et al., 1994; Takehara et al., 2016). Similar to the case in cells infected by the WT strain, infection with the Δ*plc* mutant induced caspase-1 activation and cleavage/release of IL-1β (Figures 2B,C). On the other hand, infection with the mutants Δ*pfo* and Δ*plc*Δ*pfo* was not associated with any activation of caspase-1 or cleavage/release of IL-1β (Figures 2B,C). Consistent with these findings, no release of LDH was observed from the macrophages infected with the Δ*pfo* and Δ*plc*Δ*pfo* mutants, whereas comparable levels of LDH were detected in the medium of the cells infected with the WT and Δ*plc* mutant (Figure 2D). These data clearly indicate that PFO is an essential bacterial factor for triggering inflammasome activation in macrophages.

### PFO Triggers Inflammasome Activation via the NLRP3-ASC Axis

**FIGURE 3:**
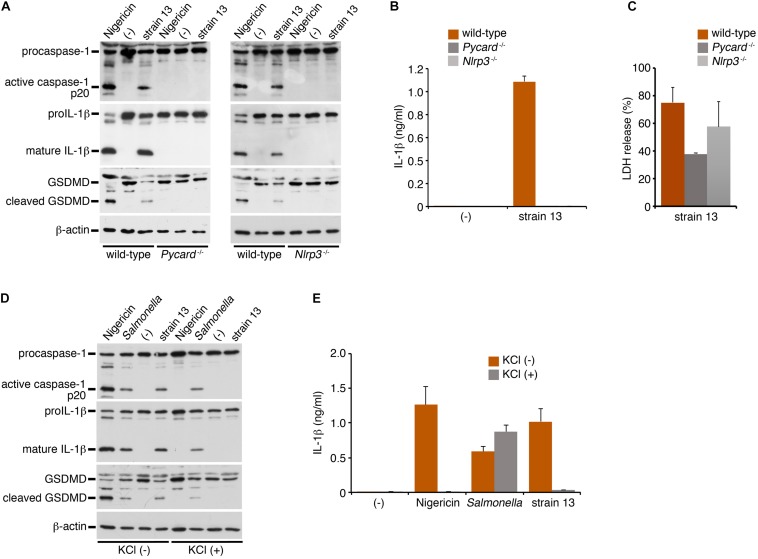
*Clostridium perfringens* triggers inflammasome activation through the NLRP3-ASC axis. BMDMs from wild-type, NLRP3- (*Nlrp3*^–/–^)- or ASC- (*Pycard*^–/–^) deficient mice were primed with LPS for 2 h and infected with WT *C. perfringens* (strain 13) for 1 h, or treated with 10 μM nigericin for 30 min as positive controls **(A,D,E)**. The BMDMs were infected with *C. perfringens*, an SPI-2-deficient mutant of *Salmonella* for 1 h or treated with nigericin in the presence or absence of KCl (130 mM) **(D,E)**. **(A,D)** The activation of caspase-1, and cleavage of IL-1β or GSDMD in the infected BMDMs were analyzed by immunoblotting with anti-caspase-1 (p20), anti-IL-1β or anti-GSDMD antibody, respectively. **(B,E)** IL-1β release from the infected BMDMs into the culture supernatants at 1 hpi was analyzed by an ELISA. Data are presented as the means ± SD of triplicate samples. **(C)** Culture supernatants of the infected BMDMs were analyzed for LDH release at 1 hpi.

NLRP3 is a member of the NLR family of proteins, and is known to mediate caspase-1 activation and IL-1β release via formation of a protein complex called inflammasome with the adaptor protein, apoptosis-associated speck-like protein containing a CARD domain (ASC, also known as Pycard) (Higa et al., 2013; Elliott and Sutterwala, 2015). Bacterial pore-forming toxins such as PFO are known to trigger NLRP3-mediated inflammasome activation. To explore whether this is the mechanism responsible for caspase-1 activation by the PFO of *C. perfringens*, NLRP3- or ASC (Pycard)-deficient BMDMs were infected with WT *C. perfringens*, followed by examination of the status of caspase-1 activation and IL-1β release. As compared to the wild-type BMDMs, caspase-1 activation and IL-1β cleavage/release by *C. perfringens* were completely abolished in both the NLRP3- and ASC-deficient BMDMs (Figures 3A,B), as in the nigericin-treated cells as the positive control. These results indicate that the inflammasome activation induced by the PFO produced by *C. perfringens* is dependent on the NLRP3-ASC axis. LDH release from infected ASC-deficient BMDMs but not NLRP3-deficient cells was slightly decreased but not abolished (Figure 3C), suggesting that necrotic cell death of macrophages by PFO is partly affected by ASC but not dependent on NLRP3-ASC-mediated inflammasome activation. NLRP3-mediated inflammasome activation is controlled by several pathways. As one such major pathway, reduction of intracellular potassium by bacterial pore-forming toxins is known to trigger inflammasome activation. High extracellular potassium concentrations are known to inhibit NLRP3-mediated inflammasome activation (Petrilli et al., 2007). To obtain further evidence in support of our finding of NLRP3-mediated inflammasome activation by PFO, wild-type BMDMs were infected with *C. perfringens* in the presence or absence of 130 mM KCl. As shown in Figures 3D,E, high extracellular potassium concentrations suppressed caspase-1 activation and cleavage/release of IL-1β induced by *C. perfringens* and nigericin, but not that induced by a *Salmonella* mutant, which induces NLRC4-mediated activation (Liu et al., 2014). These data suggest that NLRP3 recognizes K^+^ efflux triggered by the PFO produced by *C. perfringens*.

### The Infection of Myocytes With *C. perfringens* Does Not Support Inflammasome Activation

**FIGURE 4:**
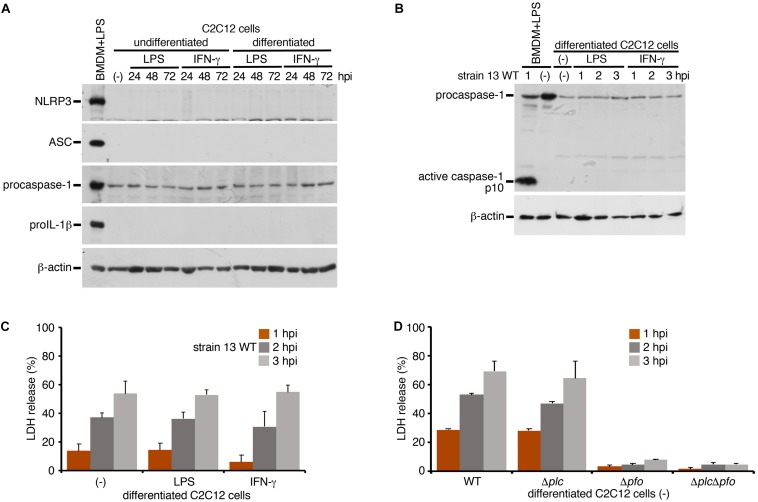
Inflammasome activation is not induced in the infected skeletal myocytes. Mouse C2C12 myoblasts or the differentiated myocytes using horse serum was treated with LPS (1 μg/ml) or IFN-γ (100 ng/ml) for 24 to 72 h. **(A)** The expression of NLRP3, ASC, procaspase-1, and proIL-1β in the cells were analyzed by immunoblotting with anti-NLRP3, anti-caspase-1 (p10) or anti-IL-1β antibody, respectively. **(B,D)** The differentiated myocytes were treated with LPS or IFN-γ for 48 h, and infected with WT *C. perfringens* (strain 13). The activation of caspase-1 was analyzed by immunoblotting **(B)**. **(C)** Culture supernatants of the infected myocytes were analyzed for LDH release at 1 to 3 hpi. **(D)** The differentiated myocytes were infected with WT *C. perfringens*, and/or any of its isogenic mutants, Δ*plc*, Δ*pfo* and Δ*plc*Δ*pfo*, and analyzed the LDH release from infected cells. Data are presented as the means ± SD of triplicate samples.

To examine whether inflammasome activation is also induced in infected myocytes, we used myocytes (myotubes) differentiated from mouse myoblast C2C12 cells in the presence of 2% horse serum. A previous report has shown that the stimulation with IFN-γ induced the activation of NLRP3 inflammasome in myocytes with enhancing expression level of NLRP3 or ASC (Ding et al., 2018). We examined the protein level of NLRP3 or ASC upon stimulation with LPS or IFN-γ under the undifferentiated or differentiated condition. However, we observed no expression of these proteins in myocytes in any conditions compared to BMDM as a control (Figure 4A). On the other hand, the expression of procaspase-1 was detected. Although the discrepancy between previous report (Ding et al., 2018) and our results may be attributable to different experimental conditions and cells used, our data suggest that, even though the stimulation with LPS or IFN-γ, it is unlikely to be able to support inflammasome activation. The differentiated myocytes were infected with the WT *C. perfringens* and examined the caspase-1 activation. In contrast to the infected BMDM, activated caspase-1 was not detected in infected myocytes (Figure 4B). NLRC4-mediated inflammasome activation was also examined using *Salmonella* mutant infection. Similar as *C. perfringens* infection, no caspase-1 activation was detected (data not shown). The LDH after infection with WT *C. perfringens* was equally released regardless of the presence or absence of LPS or IFN-γ (Figure 4C). Also, similar to the case in the infected BMDMs, PFO but not α-toxin triggered LDH release in the infected myocytes (Figure 4D). These data suggest that PFO produced by *C. perfringens* induces necrotic cell death but does not trigger inflammasome activation in the infected myocytes.

### PFO Is Involved in the Induction of Myonecrosis of the Infected Muscle Tissue in Mice Through Triggering NLRP3-Mediated Inflammasome Activation

**FIGURE 5:**
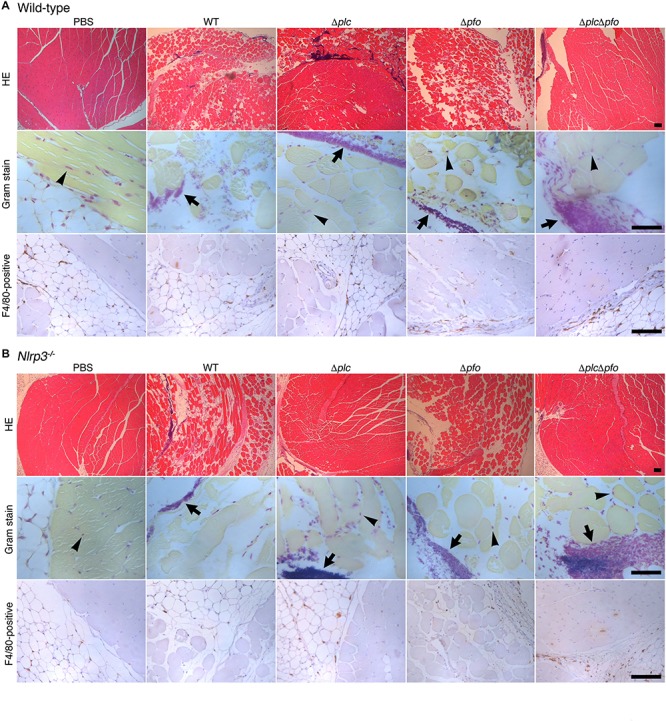
PFO and NLRP3 are involved in part in the progression of myonecrosis in the muscle tissues infected by *Clostridium perfringens*. C57BL/6 wild-type **(A)** or NLRP3-deficient mice **(B)** (wild = type, *n* = 10 and gene- knockout mice, *n* = 8 for infection with WT *C. perfringens*, and *n* = 5 for infection with other mutants or incubation with PBS) were intramuscularly infected with WT *C. perfringens* and/or any of its isogenic mutants. The sections of infected muscle tissues at 6 hpi were analyzed by HE, Gram stainings and immunostainings with anti-F4/80 antibody to detect macrophage lineage. Representative data are shown. Black arrows indicate the infecting bacteria and black arrow heads indicate nonspecific staining to nuclei of myocytes. Bars, 100 μm.

**FIGURE 6:**
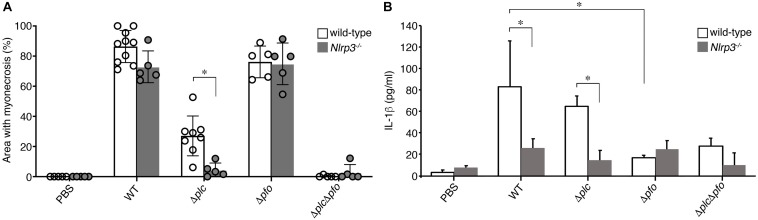
NLRP3-mediated myonecrosis and IL-1β production. **(A)** Quantitative analysis of the area of myonecrosis from the data in **(A,B)** in Figure 5. **(B)** IL-1β production in the infected muscle tissues analyzed by an ELISA. Data are presented as the means ± SD and compared using a *t*-test (^∗^*p* < 0.05).

Previous reports have suggested the synergistic effects of α-toxin and PFO in the development of the typical pathological features of *C. perfringens* infection, that is, myonecrosis, platelet-leukocyte accumulation, leukostasis, and reduction of microvascular perfusion (Ellemor et al., 1999; Awad et al., 2001; Hickey et al., 2008). However, there are no reports of quantitative evaluation of the synergistic effects of α-toxin and PFO in the induction of necrosis of the infected muscle tissue. Therefore, we performed quantitative analysis to determine the areas of myonecrosis after intramuscular infection of mice with WT *C. perfringens* and/or any of the isogenic mutants. We initially examined the bacterial loads in the infected muscle tissues after 6 hpi. No significant difference in the bacterial load was observed between the different bacterial strains or genotypes of the mice (data not shown). Necrosis and dissociation of muscle tissue were observed in 86% of the total muscle tissue area in the WT-infected mice, and in a lesser area (76%) in the PFO mutant (Δ*pfo*)-infected mice. On the other hand, infection with the α-toxin mutant (Δ*plc*) induced myonecrosis of 25% of the infected muscle area (Figure 5A). These results suggest that although α-toxin acts as a major player in the induction of myonecrosis, PFO is also involved in part in the disease progression. The infecting Gram-positive bacilli were observed in the peripheral areas of the infected muscle by Gram-staining (Figure 5A). To examine whether NLRP3-mediated inflammasome activation is involved in the myonecrosis induced by PFO, NLRP3-deficient mice were infected with the series *C. perfringens* strains. The area of myonecrosis after infection was significantly decreased in the case of Δ*plc* infection, but not in the case of infection with WT *C. perfringens* or the Δ*pfo* mutant (Figures 5B, 6A), suggesting that the limited action of PFO in inducing myonecrosis is clearly dependent on NLRP3. Next, we immunostained the sectioned slices of infected muscle tissues with anti-F4/80 antibody to detect macrophage lineage. As shown in Figures 5A,B, some F4/80-positive cells were detected in interstitial spaces of muscle tissue and fascial layer in both of wild-type and NLRP3-deficient mice, suggesting the possibility that these macrophages play a role in inflammasome activation and IL-1β induction. We then quantified the levels of IL-1β in infected mouse muscle tissues and observed the small but significant amounts of IL-1β were produced in PFO- and NLRP3-dependent manner (Figure 6B). Furthermore, we attempted to quantify the proinflammatory cytokine levels of IL-6 and TNF-α in the muscle tissues infected with the series of *C. perfringens* strains by ELISA. However, the levels of the cytokines were very low (less than tens of pg/ml) and varied among the mice (data not shown).

## Discussion

*Clostridium perfringens*, mainly type A strains, cause a fast-spreading and potentially life-threatening disease called gas gangrene. The bacterium produces several virulence factors, including α-toxin and PFO, which are involved in the progression of myonecrosis, the characteristic feature of gas gangrene. Although these two toxins are necessary to induce the myonecrosis, our understanding of the molecular mechanism of the infection process is still limited. In the present study, we demonstrated *in vitro* that PFO, but not α-toxin, clearly triggers NLRP3-mediated inflammasome activation in infected macrophages. Using a mouse model of muscle infection, we demonstrated that the main bacterial factor that induces myonecrosis is α-toxin, although PFO also participates in the progression of the disease in part. We further demonstrated that PFO-mediated myonecrosis is dependent on NLRP3.

We demonstrated that IL-1β production is induced by NLRP3-mediated inflammasome activation mediated by PFO *in vitro*. Since myonecrosis induced by infection with the α-toxin mutant (Δ*plc*) was inhibited in the NLRP3-deficient mice, PFO-driven myonecrosis was shown to be NLRP3-dependent. Also, PFO-mediated IL-1β production was detected in infected muscle tissues *in vivo* and the induction was NLRP3-dependent manner, but it is unclear which cells are triggered NLRP3-mediated inflammasome in infected muscle tissues. Since some F4/80-positive macrophage lineage cells were detected in interstitial spaces of muscle tissue and fascial layer, it raises the possibility that these macrophages cause inflammasome activation and produce IL-1β. On the other hand, in infected myocytes differentiated from of C2C12 myoblasts, neither activation of inflammasome nor production of IL-1β was detected, but necrosis of the cells was observed. Our data suggested that infected myocytes may not play a role in triggering of inflammasome activation *in vivo*. On the other hand, it remains to be elucidated whether IL-1β induced by inflammasome activation is responsible for the PFO-mediated myonecrosis. It is possible that the infected cells that undergo pyroptosis or cell death as a result of inflammasome activation are involved in some part in triggering necrosis of the infected muscle tissue (O’Brien and Melville, 2004; Park et al., 2004). Alternatively, as a result of IL-1β induction via inflammasome activation induced by PFO, neutrophils may be recruited around the infected tissues and potentially contribute to reduction in microvascular perfusion. Taken together, our data suggest that proinflammatory responses triggered by NLRP3-mediated inflammasome are involved in the progression of myonecrosis induced by PFO. Meanwhile, α-toxin triggers myonecrosis by essentially different mechanisms, in the absence of obvious inflammation, such as inflammasome activation or IL-1β induction.

The biological and enzymatic activities of α-toxin and PFO differ. Nevertheless, in the mouse model of muscle infection, the pathological features induced by the two toxins appeared similar. α-toxin has phospholipase-C activity which hydrolyzes phosphatidylcholine and sphingomyelin to produce ceramide and diacylglycerol, a second messenger in the cells, and affects many different cellular pathways. The phospholipase-C activity of α-toxin also activates arachidonic acid cascades, which are involved in muscle contraction and platelet aggregation, resulting in decreased perfusion (Navarro et al., 2018). On the other hand, addition of a lytic concentration of α-toxin resulted in necrosis via degradation of the plasma membrane in several cultured cell lines (Flores-Diaz et al., 2005). The induction of apoptosis via activation of cellular signals by α-toxin has been also suggested (Navarro et al., 2018). However, in our experiments involving infection with isogenic mutants, we did not find any necrotic or apoptotic features induced by α-toxin in the infected macrophages. On the other hand, the biological activity of PFO may be, to some extent, more easily understandable. Our data showed that the pore-forming action of PFO triggers inflammasome activation via NLRP3, which is necessary for the induction of myonecrosis by PFO. At least, the possibility that the pore-forming activity *per se* of PFO directly induces myonecrosis was excluded. NLRP3-mediated necrosis or induction of IL-1β appears to be involved in the myonecrosis induced by PFO.

In summary, we investigated the functional roles of inflammasome activation in tissues infected with *C. perfringens*. The bacteria trigger caspase-1 activation and consequently, IL-1β release, through NLRP3-mediated inflammasome activation by PFO in the infected macrophages. Furthermore, we demonstrated, for the first time, that the myonecrosis induced by PFO was dependent on NLRP3. Our data suggest that the PFO produced by *C. perfringens* activates inflammasomes via NLRP3 to induce myonecrosis in the infected muscle tissues. Although it was not possible to clarify the molecular mechanisms underlying the inflammasome-mediated myonecrosis driven by PFO, our data shed light on PFO-mediated inflammatory responses in the host to *C. perfringens* infection.

## Data Availability Statement

The datasets generated for this study are available on request to the corresponding author.

## Ethics Statement

The animal study was reviewed and approved by the Institutional Animal Care and Use Committee of Tokyo Medical and Dental University.

## Author Contributions

KY, TO, and TS conceived and designed the experiments. KY and HA performed the experiments. KY, TO, SS, TI, and TS analyzed the data. HA, SS, KO, RK-D, MH, and TI contributed reagents, materials, and analysis tools. KY and TS prepared the manuscript.

## Conflict of Interest

The authors declare that the research was conducted in the absence of any commercial or financial relationships that could be construed as a potential conflict of interest.

## Footnotes

1 http://rsb.info.nih.gov/ij//

## References

[B1] Akira S. Uematsu S. Takeuchi O. (2006). Pathogen recognition and innate immunity. *Cell* 124 783–801. 10.1016/j.cell.2006.02.015 16497588

[B2] Awad M. M. Bryant A. E. Stevens D. L. Rood J. I. (1995). Virulence studies on chromosomal alpha-toxin and theta-toxin mutants constructed by allelic exchange provide genetic evidence for the essential role of alpha-toxin in *Clostridium perfringens*-mediated gas gangrene. *Mol. Microbiol.* 15 191–202. 10.1111/j.1365-2958.1995.tb02234.x 7746141

[B3] Awad M. M. Ellemor D. M. Boyd R. L. Emmins J. J. Rood J. I. (2001). Synergistic effects of alpha-toxin and perfringolysin O in *Clostridium perfringens*-mediated gas gangrene. *Infect. Immun.* 69 7904–7910. 10.1128/IAI.69.12.7904-7910.2001 11705975 PMC98889

[B4] Bryant A. E. Bayer C. R. Aldape M. J. Wallace R. J. Titball R. W. Stevens D. L. (2006). *Clostridium perfringens* phospholipase C-induced platelet/leukocyte interactions impede neutrophil diapedesis. *J. Med. Microbiol.* 55(Pt 5), 495–504. 10.1099/jmm.0.46390-0 16585634

[B5] Bryant A. E. Chen R. Y. Nagata Y. Wang Y. Lee C. H. Finegold S. (2000a). Clostridial gas gangrene. I. Cellular and molecular mechanisms of microvascular dysfunction induced by exotoxins of *Clostridium perfringens*. *J. Infect. Dis.* 182 799–807. 10.1086/315756 10950774

[B6] Bryant A. E. Chen R. Y. Nagata Y. Wang Y. Lee C. H. Finegold S. (2000b). Clostridial gas gangrene. II. Phospholipase C-induced activation of platelet gpIIbIIIa mediates vascular occlusion and myonecrosis in *Clostridium perfringens* gas gangrene. *J. Infect. Dis.* 182 808–815. 10.1086/315757 10950775

[B7] Bryant A. E. Stevens D. L. (1996). Phospholipase C and perfringolysin O from *Clostridium perfringens* upregulate endothelial cell-leukocyte adherence molecule 1 and intercellular leukocyte adherence molecule 1 expression and induce interleukin-8 synthesis in cultured human umbilical vein endothelial cells. *Infect. Immun.* 64 358–362. 8557365 10.1128/iai.64.1.358-362.1996 PMC173769

[B8] Bryant A. E. Stevens D. L. (2010). Clostridial myonecrosis: new insights in pathogenesis and management. *Curr. Infect. Dis. Rep.* 12 383–391. 10.1007/s11908-010-0127-y 21308521

[B9] Bunting M. Lorant D. E. Bryant A. E. Zimmerman G. A. McIntyre T. M. Stevens D. L. (1997). Alpha toxin from *Clostridium perfringens* induces proinflammatory changes in endothelial cells. *J. Clin. Invest.* 100 565–574. 10.1172/JCI119566 9239403 PMC508223

[B10] Ding M. Huang T. Zhu R. Gu R. Shi D. Xiao J. (2018). Immunological behavior analysis of muscle cells under IFN-gamma stimulation *in vitro* and *in vivo*. *Anat. Rec.* 301 1551–1563. 10.1002/ar.23834 29669192

[B11] Ellemor D. M. Baird R. N. Awad M. M. Boyd R. L. Rood J. I. Emmins J. J. (1999). Use of genetically manipulated strains of *Clostridium perfringens* reveals that both alpha-toxin and theta-toxin are required for vascular leukostasis to occur in experimental gas gangrene. *Infect. Immun.* 67 4902–4907. 10456947 10.1128/iai.67.9.4902-4907.1999 PMC96825

[B12] Elliott E. I. Sutterwala F. S. (2015). Initiation and perpetuation of NLRP3 inflammasome activation and assembly. *Immunol. Rev.* 265 35–52. 10.1111/imr.12286 25879282 PMC4400874

[B13] Flores-Diaz M. Alape-Giron A. Clark G. Catimel B. Hirabayashi Y. Nice E. (2005). A cellular deficiency of gangliosides causes hypersensitivity to *Clostridium perfringens* phospholipase C. *J. Biol. Chem.* 280 26680–26689. 10.1074/jbc.M500278200 15919667

[B14] Hickey M. J. Kwan R. Y. Awad M. M. Kennedy C. L. Young L. F. Hall P. (2008). Molecular and cellular basis of microvascular perfusion deficits induced by *Clostridium perfringens* and *Clostridium septicum*. *PLoS Pathog.* 4:e1000045. 10.1371/journal.ppat.1000045 18404211 PMC2275794

[B15] Higa N. Toma C. Nohara T. Nakasone N. Takaesu G. Suzuki T. (2013). Lose the battle to win the war: bacterial strategies for evading host inflammasome activation. *Trends Microbiol.* 21 342–349. 10.1016/j.tim.2013.04.005 23712018

[B16] Keese M. Nichterlein T. Hahn M. Magdeburg R. Karaorman M. Back W. (2003). Gas gangrene pyaemia with myocardial abscess formation–fatal outcome from a rare infection nowadays. *Resuscitation* 58 219–225. 10.1016/s0300-9572(03)00121-7 12909385

[B17] Lamkanfi M. Dixit V. M. (2017). In retrospect: the inflammasome turns 15. *Nature* 548 534–535. 10.1038/548534a 28858314

[B18] Liu T. Yamaguchi Y. Shirasaki Y. Shikada K. Yamagishi M. Hoshino K. (2014). Single-cell imaging of caspase-1 dynamics reveals an all-or-none inflammasome signaling response. *Cell Rep.* 8 974–982. 10.1016/j.celrep.2014.07.012 25127135

[B19] Low L. Y. Harrison P. F. Gould J. Powell D. R. Choo J. M. Forster S. C. (2018). Concurrent host-pathogen transcriptional responses in a *Clostridium perfringens* murine myonecrosis infection. *mBio* 9:e473-18. 10.1128/mBio.00473-18 29588405 PMC5874911

[B20] Mollby R. Holme T. Nord C. E. Smyth C. J. Wadstrom T. (1976). Production of phospholipase C (alpha-toxin), haemolysins and lethal toxins by *Clostridium perfringens* types A to D. *J. Gen. Microbiol.* 96 137–144. 10.1099/00221287-96-1-137 10344

[B21] Navarro M. A. McClane B. A. Uzal F. A. (2018). Mechanisms of action and cell death associated with *Clostridium perfringens* toxins. *Toxins* 10:E212. 10.3390/toxins10050212 29786671 PMC5983268

[B22] O’Brien D. K. Melville S. B. (2004). Effects of *Clostridium perfringens* alpha-toxin (PLC) and perfringolysin O (PFO) on cytotoxicity to macrophages, on escape from the phagosomes of macrophages, and on persistence of C. *perfringens in host tissues*. *Infect. Immun.* 72 5204–5215. 10.1128/IAI.72.9.5204-5215.2004 15322015 PMC517428

[B23] Ochi S. Miyawaki T. Matsuda H. Oda M. Nagahama M. Sakurai J. (2002). Clostridium perfringens alpha-toxin induces rabbit neutrophil adhesion. *Microbiology* 148(Pt 1), 237–245. 10.1099/00221287-148-1-237 11782516

[B24] Oda M. Kihara A. Yoshioka H. Saito Y. Watanabe N. Uoo K. (2008). Effect of erythromycin on biological activities induced by *Clostridium perfringens* alpha-toxin. *J. Pharmacol. Exp. Ther.* 327 934–940. 10.1124/jpet.108.143677 18794379

[B25] Oda M. Shiihara R. Ohmae Y. Kabura M. Takagishi T. Kobayashi K. (2012). *Clostridium perfringens* alpha-toxin induces the release of IL-8 through a dual pathway via TrkA in A549 cells. *Biochim. Biophys. Acta* 1822 1581–1589. 10.1016/j.bbadis.2012.06.007 22721959

[B26] Park J. M. Ng V. H. Maeda S. Rest R. F. Karin M. (2004). Anthrolysin O and other gram-positive cytolysins are toll-like receptor 4 agonists. *J. Exp. Med.* 200 1647–1655. 10.1084/jem.20041215 15611291 PMC2211988

[B27] Petrilli V. Papin S. Dostert C. Mayor A. Martinon F. Tschopp J. (2007). Activation of the NALP3 inflammasome is triggered by low intracellular potassium concentration. *Cell Death Differ.* 14 1583–1589. 10.1038/sj.cdd.4402195 17599094

[B28] Shi J. Gao W. Shao F. (2017). Pyroptosis: gasdermin-mediated programmed necrotic cell death. *Trends Biochem. Sci.* 42 245–254. 10.1016/j.tibs.2016.10.004 27932073

[B29] Shimizu T. Ba-Thein W. Tamaki M. Hayashi H. (1994). The virR gene, a member of a class of two-component response regulators, regulates the production of perfringolysin O, collagenase, and hemagglutinin in *Clostridium perfringens*. *J. Bacteriol.* 176 1616–1623. 10.1128/jb.176.6.1616-1623.1994 8132455 PMC205246

[B30] Shimizu T. Ohtani K. Hirakawa H. Ohshima K. Yamashita A. Shiba T. (2002). Complete genome sequence of *Clostridium perfringens*, an anaerobic flesh-eater. *Proc. Natl. Acad. Sci. U.S.A.* 99 996–1001. 10.1073/pnas.022493799 11792842 PMC117419

[B31] Songer J. G. (1996). Clostridial enteric diseases of domestic animals. *Clin. Microbiol. Rev.* 9 216–234. 10.1128/cmr.9.2.216 8964036 PMC172891

[B32] Takehara M. Takagishi T. Seike S. Ohtani K. Kobayashi K. Miyamoto K. (2016). *Clostridium perfringens* alpha-toxin impairs innate immunity via inhibition of neutrophil differentiation. *Sci. Rep.* 6:28192. 10.1038/srep28192 27306065 PMC4910053

[B33] Uzal F. A. Freedman J. C. Shrestha A. Theoret J. R. Garcia J. Awad M. M. (2014). Towards an understanding of the role of *Clostridium perfringens* toxins in human and animal disease. *Future Microbiol.* 9 361–377. 10.2217/fmb.13.168 24762309 PMC4155746

[B34] Uzal F. A. Vidal J. E. McClane B. A. Gurjar A. A. (2010). *Clostridium perfringens* toxins involved in mammalian veterinary diseases. *Open Toxinol. J.* 2 24–42. 10.2174/1875414701003010024 24511335 PMC3917546

