# Supplementary figures and images for: Inflammasome Activation Induced by Perfringolysin O of Clostridium perfringens and Its Involvement in the Progression of Gas Gangrene
Source: Front Microbiol. 2019 Oct 25;10:2406. doi: 10.3389/fmicb.2019.02406 (PMC6823607; doi:10.3389/fmicb.2019.02406)

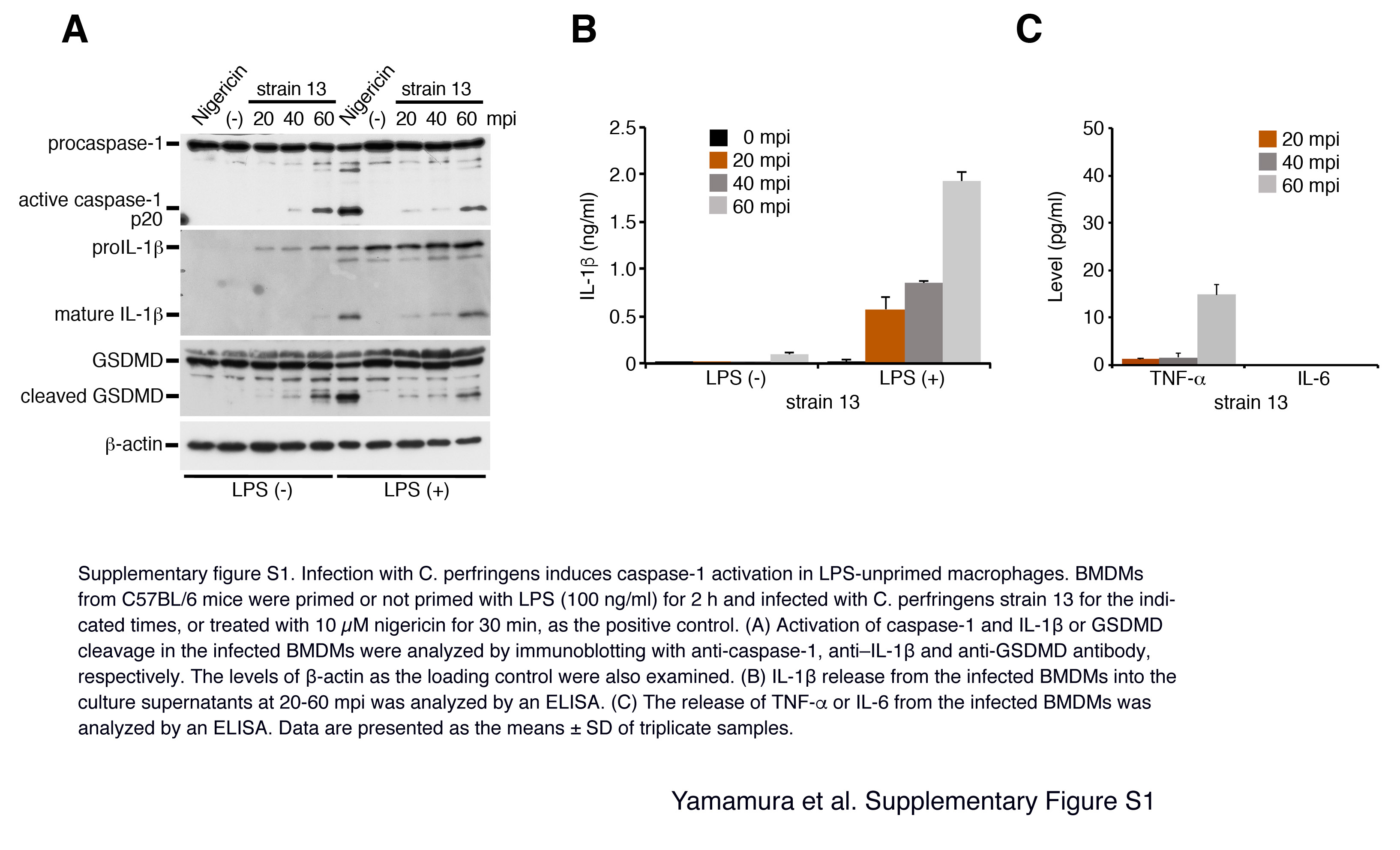

Supplement: Supplementary file 1 [file Image_1.JPEG]
